# Multiple testing correction in linear mixed models

**DOI:** 10.1186/s13059-016-0903-6

**Published:** 2016-04-01

**Authors:** Jong Wha J. Joo, Farhad Hormozdiari, Buhm Han, Eleazar Eskin

**Affiliations:** Bioinformatics IDP, University of California, Los Angeles, CA USA; Computer Science Department, University of California, Los Angeles, CA USA; Department of Convergence Medicine, University of Ulsan College of Medicine & Asan Institute for Life Sciences, Asan Medical Center, Seoul, 138-736 Republic of Korea; Department of Human Genetics, University of California, Los Angeles, CA USA

## Abstract

**Background:**

Multiple hypothesis testing is a major issue in genome-wide association studies (GWAS), which often analyze millions of markers. The permutation test is considered to be the gold standard in multiple testing correction as it accurately takes into account the correlation structure of the genome. Recently, the linear mixed model (LMM) has become the standard practice in GWAS, addressing issues of population structure and insufficient power. However, none of the current multiple testing approaches are applicable to LMM.

**Results:**

We were able to estimate per-marker thresholds as accurately as the gold standard approach in real and simulated datasets, while reducing the time required from months to hours. We applied our approach to mouse, yeast, and human datasets to demonstrate the accuracy and efficiency of our approach.

**Conclusions:**

We provide an efficient and accurate multiple testing correction approach for linear mixed models. We further provide an intuition about the relationships between per-marker threshold, genetic relatedness, and heritability, based on our observations in real data.

**Electronic supplementary material:**

The online version of this article (doi:10.1186/s13059-016-0903-6) contains supplementary material, which is available to authorized users.

## Background

Genome-wide association studies (GWAS) have discovered many variants implicated in complex traits in studies of both humans [1–8] and model organisms [9–16]. In GWAS, both genetic information on variants spread throughout the genome and phenotypic information are collected from a population. The correlation between the genetic information at each variant, referred to as the genotype, and the phenotypic information is assessed to identify the set of variants associated with the trait of interest. GWAS now are routinely performed on tens of thousands of individuals and millions of genetic variants.

One of the major challenges in GWAS is multiple hypothesis testing. Because each GWAS involves computing up to millions of statistical tests, the *p* value threshold for significance, referred to as the per-marker threshold, must be adjusted to control the overall false positive rate. The Bonferroni correction [17] assumes independence among the association tests. However, there is a substantial degree of correlation between the association statistics due to a phenomenon called linkage disequilibrium [18], which renders the Bonferroni correction too conservative [19]. The permutation test [20], which samples the null distribution of statistics by repeatedly permuting the phenotypes and computing the association statistics for each permutation, is considered to be the gold standard because it accurately accounts for the correlation structure of the genome at the expense of computational cost. Several strategies aimed at speeding up the computational cost of the permutation test have recently been developed [21–24].

Recently, the linear mixed model (LMM) [25–31] has become the standard practice for performing GWAS. The LMM can address two important challenges in GWAS: population structure and insufficient power. Population structure refers to a complex relatedness structure among individuals, which can generate false positives or spurious associations when utilizing traditional association study techniques [26, 27]. LMM approaches can avoid these false positives by explicitly modeling these genetic relationships [26, 27, 29–33]. Moreover, even when there is no population structure, LMM can increase the statistical power of GWAS [31, 34, 35]. Due to these desirable properties, LMM has become a widely used method in current GWAS [36–40].

However, the current approaches for multiple hypothesis testing correction cannot be applied to LMM. Even the gold standard, the permutation test, is not applicable to LMM, because the underlying idea is that each permutation represents a sample from the null distribution. This is not the case in LMM, because the phenotypes have a covariance structure induced by the complex patterns of relatedness among the individuals. Unfortunately, to date no available approach can correct for multiple testing in LMM, because almost all known multiple testing correction approaches are based on the permutation test and aim only to increase the efficiency of the permutation test [21–24, 41]. By performing simulations, we demonstrated that the multiple testing burden changes with heritability, and that the permutation test inaccurately corrects for the multiple testing when heritability is non-zero.

In this paper, we first set up the gold standard approach for multiple testing correction in LMM. Our approach is a bootstrapping resampling approach that is the equivalent of the permutation test for LMM. Specifically, our parametric bootstrapping approach samples randomized null phenotypes from the distribution fitted by LMM. This approach straightforwardly accounts for the effect of between-individual genetic relatedness on phenotypes. However, like the permutation test, this approach is computationally expensive due to the large number of resamplings, and is therefore only suitable for small datasets.

To address this issue, we developed a new approach called multiple testing in transformed space (MultiTrans), which can efficiently correct for multiple testing for LMM. To approximate the results of parametric bootstrapping efficiently, we employ a strategy that directly samples statistics instead of sampling phenotypes. Both sampling phenotypes in bootstrapping and sampling of statistics in our new approach involve sampling from a multivariate normal distribution (MVN). However, the sampling of statistics is much more efficient because the time complexity of the sampling procedure is independent of the number of individuals. To obtain the covariance matrix of the MVN for statistics, previous strategies [21–24] that directly use the genotype correlation structure as the covariance matrix cannot be applied, because such a relationship no longer holds under LMM. Therefore, we developed a new approach to overcome this challenge, which transforms genotype dosages into a space where the phenotypic correlation between related individuals can be accounted for. Finally, to reduce computational cost in GWAS where linkage disequilibrium is expected to be local, we apply the sliding-window-based sampling approach [24]. We applied our approach to the Hybrid Mouse Diversity Panel (HMDP) dataset [11], a yeast dataset [10] and the HapMap dataset [42]; the results demonstrate that our method can perform multiple hypothesis correction as accurately as parametric bootstrapping, while reducing the time required from months to hours. Applying our approach to a number of different phenotypes in these real datasets also provided an intuition that the per-marker threshold depends on both the heritability of the trait and the genetic relatedness between individuals. We expect that our method will be widely used to obtain correct per-marker threshold in future studies utilizing LMM.

## Results

### Overview of the method

In multiple testing correction, our goal is to find the per-marker threshold that gives an overall false positive rate of *α*. Let us assume the following linear model:

(1) $$ Y=\mu{\mathbf{1_{n}}}+X_{i}\beta_{i}+\mathbf{e}.   $$

Here, *n* is the number of individuals, *μ* is the mean of the phenotypic values, 1_*n*_ is a vector of *n* ones, *Y* is a vector of length *n* with the phenotypic values, *X*_*i*_ is a vector of length *n* with the genotypic values of the *i*th marker, *β*_*i*_ is the coefficient of the *i*th marker, and **e** is a vector of length *n* sampled from $\mathcal {N}(0, \sigma ^{2}\mathbf {I})$ accounting for the residual errors. Let *S*_*i*_ and *S*_*j*_ be the test statistics for the *i*th and *j*th markers under the linear model, accordingly. Under the assumption of a linear model (Eq. 1), we can derive the equality between the covariance of the two statistics, Cov(*S*_*i*_,*S*_*j*_), and the correlation of the genotypes, *r*_*ij*_, as follows:

(2) $$ \text{Cov}(S_{i}, S_{j})=\frac{{X_{i}^{T}}X_{j}}{\sqrt{{X_{i}^{T}}X_{i}}\sqrt{{X_{j}^{T}}X_{j}}} = \text{Cor}\left(X_{i}, X_{j}\right)\equiv r_{ij}.   $$

The derivation of this equality is described in detail in section “Methods”. This property has been reported in previous studies [24, 43, 44].

**Fig. 1:**
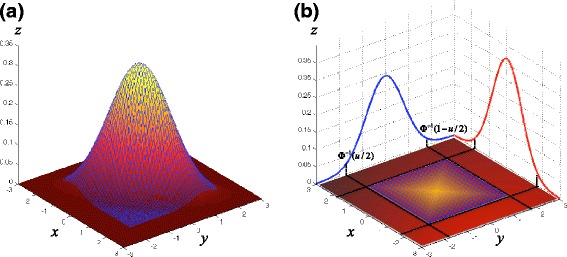
Probability density function of a bivariate MVN at two markers under the null hypothesis. Image (**b**) when we project the MVN (**a**) into the *xy* space

Let *m* be the number of markers and *Σ* be the *m*×*m* covariance matrix between the statistics whose (*i*, *j*)th element is *Σ*_*i*,*j*_=Cov(*S*_*i*_,*S*_*j*_). According to the multivariate central limit theorem [45], when *n* is large, the vector of statistics (*S*_1_,…,*S*_*m*_) asymptotically follows a MVN with mean 0 and variance *Σ*. Figure 1a shows a probability density function of a bivariate normal distribution at two markers under the null hypothesis. The area outside the meshed rectangle region shows the critical region under the null hypothesis in which, if a *p* value falls within this region, the null hypothesis is rejected. Figure 1b shows the image when we project the MVN in Fig. 1a into the *xy* space. Let *u* be the pointwise *p* value that is shown as each point in the MVN. The four corners of the shaded rectangle are (*Φ*^−1^(*u*/2),*Φ*^−1^(*u*/2)), (*Φ*^−1^(1−*u*/2),*Φ*^−1^(*u*/2)), (*Φ*^−1^(*u*/2),*Φ*^−1^(1−*u*/2)) and (*Φ*^−1^(1−*u*/2),*Φ*^−1^(1−*u*/2)), where *Φ* is the cumulative density function of the standard normal distribution. Let *p*_*α*_ be the outside-rectangle probability in Fig. 1b. Then, given an overall significance level *α*, the per-marker threshold is approximated by searching for the pointwise *p* value *u* whose *p*_*α*_ is *α*. Utilizing the equality in Eq. 2, the covariance matrix of the MVN could be estimated as *Σ*={*r*_*ij*_} under the linear model (Eq. 1).

However, in LMM, the properties in Eq. 2 are no longer valid. Let us assume the following LMM:

(3) $$ Y=\mu\mathbf{1_{n}}+X_{i}{\beta_{i}^{M}}+\mathbf{g}+\mathbf{e}.   $$

Here, ${\beta _{i}^{M}}$ are the coefficients of the *i*th marker under the LMM. LMM has an extra term **g** compared to the linear model (Eq. 1), which is a vector of length *n* sampled from $\mathcal {N}\left (0, {\sigma _{g}^{2}}\mathbf {K}\right)$ accounting for the effect of genetic relatedness, where **K** is a *n*×*n* kinship matrix that explains the genetic correlation between individuals. Under the LMM, $Y \sim \mathcal {N}\left (\mu \mathbf {1_{n}}+X_{i}{\beta _{i}^{M}}, {\sigma _{g}^{2}}\mathbf {K}+{\sigma _{e}^{2}}\mathbf {I}\right)$ and the equality between the covariance of statistics and correlation of genotypes in Eq. 2 is no longer valid. Let ${S_{i}^{M}}$ and ${S_{j}^{M}}$ be the test statistics under the LMM and $\hat {V}= \hat {\sigma }_{g}^{2}\mathbf {K}, +\hat {\sigma }_{e}^{2}\mathbf {I}$ be the estimated covariance matrix by fitting the data into the LMM. Then, the covariance between the statistics in Eq. 2 changes as follows:

(4) $$\begin{array}{*{20}l} \text{Cov}\left({S_{i}^{M}}, {S_{j}^{M}}\right) & = \frac{{X^{T}_{i}}\hat{V}^{-1}X_{j}}{\sqrt{{X^{T}_{i}}\hat{V}^{-1}X_{i}}\sqrt{{X^{T}_{j}}\hat{V}^{-1}X_{j}}} \end{array} $$

(5) $$\begin{array}{*{20}l} &= \text{Cor}\left(\hat{V}^{-1/2}X_{i}, \hat{V}^{-1/2}X_{j}\right)\equiv r^{M}_{ij}.  \end{array} $$

That is, the covariance is equivalent to the correlation of the genotype data that is transformed by $\hat {V}^{-1/2}$ (which is why we call our method multiple-testing in transformed space, or MultiTrans). The details of the derivation are provided in the section “Methods”. Note that the covariance of statistics of two markers that are in linkage disequilibrium with each other depends on $\hat {V}$, which in turn depends on the heritability (${\sigma ^{2}_{g}}$) of the trait. Thus, heritability affects the covariance of the statistics, which results in different per-marker thresholds. Utilizing Eq. 5, we can compute $\Sigma ^{M}=\left \{r^{M}_{ij}\right \}$ directly from genotypes and sample the test statistics from the MVN with *Σ*^*M*^ to approximate the true null distribution and find the correct per-marker threshold. To sample statistics from the MVN efficiently, we adapt a sliding-window Monte Carlo approach [24].

### Permutation is inaccurate in LMM

LMM has become one of the standard analysis methods for GWAS [25–31, 34, 35] because it can explicitly model hidden factors, such as population structure, to avoid false positives, and can also increase the statistical power of the study. However, the permutation test, which has been widely considered to be the gold standard for multiple testing, is not applicable to LMM. The underlying assumption of the permutation test is that if we permute either the genotypes or phenotypes, we can generate the null distribution of our test statistics. However, under the LMM, permutation alters correlations between the individuals specific to LMM, and the correlation is no longer explained by the permuted genotypes or the phenotypes. Thus, applying LMMs to permuted data may result in spurious statistics. Alternatively, we can generate a null distribution for LMM by utilizing parametric bootstrapping, a resampling method that samples null phenotypes from MVN based on LMM and uses them to generate the null distribution (see section “Methods” for the details of the parametric bootstrapping). A similar approach was used in a previous study of power calculation [46], and it can be thought of as the gold standard approach for LMM.

**Fig. 2:**
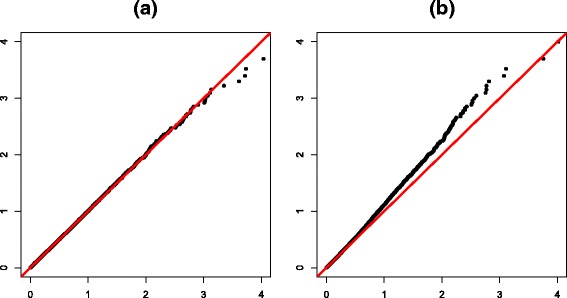
*Q*–*Q* plots of *p* values estimated from parametric bootstrapping and the permutation test for LMM under the null hypothesis. It uses 100 markers and LDL estimates from the HMDP dataset. The *x*-axis shows the quantiles of − log values of the uniform distribution and the *y*-axis shows the quantiles of − log*p* of parametric bootstrapping (**a**) and the permutation test (**b**). The *red line* is a diagonal

To show that the permutation cannot approximate the true null distribution for LMM, whereas parametric bootstrapping can do so accurately, we evaluated *p* values estimated from the permutation test and those estimated from the parametric bootstrapping for LMM under the null hypothesis. Because the HMDP dataset [11] is known to contain a significant amount of population structure [16], we used 100 genotypes and a phenotype of low-density lipoprotein (LDL) estimates from this dataset. For the permutation test, we first permuted the phenotype 10,000 times. Next, we estimated a *p* value for each genotype–phenotype pair by fitting the data to the LMM (Eq. 3) using a kinship matrix, **K**, estimated from the whole genome of the HMDP dataset. For parametric bootstrapping, we first fitted the data to the LMM and estimated its parameters, $\hat {\sigma }_{g}^{2}=0.702$ and $\hat {\sigma }_{e}^{2}=0.298$. Using these parameters, we sampled 10,000 null phenotypes from MVN with the covariance matrix, $\hat {V}=\hat {\sigma }_{g}^{2}\mathbf {K}+ \hat {\sigma }_{e}^{2}\mathbf {I}$. Then, we estimated a *p* value for each genotype–phenotype pair by fitting the data to the LMM using a kinship matrix, **K**, estimated from the whole genome of the HMDP dataset. Figure 2 shows *Q*–*Q* plots for the parametric bootstrapping (a) and the permutation test (b), which demonstrate that parametric bootstrapping can accurately approximate the null distribution for LMM. On the other hand, the permutation test yielded inflated *p* values, which demonstrates that the distribution generated from the permutation cannot be used to approximate the true null distribution for LMM.

### MultiTrans accurately approximates covariance between test statistics

As shown in the previous section, the parametric bootstrapping closely approximates the true null distribution for LMM, and can, thus, be used as the gold standard for multiple testing in LMM. MultiTrans is rooted in the idea of parametric bootstrapping. However, to approximate the results of parametric bootstrapping efficiently, MultiTrans samples statistics directly from MVN with a covariance matrix estimated from transformed genotypes. In this section, we show how accurately MultiTrans approximates the covariance matrix of test statistics using the transformation strategy (Eq. 5), by testing the difference between the empirical estimate of covariance of test statistics, $\text {Cov}\left ({S_{i}^{M}}, {S_{j}^{M}}\right)$, and the correlation of transformed genotypes, $\text {Cor}\left (\hat {V}^{-1/2}X_{i}, \hat {V}^{-1/2}X_{j}\right)$, utilizing simulated datasets.

**Fig. 3:**
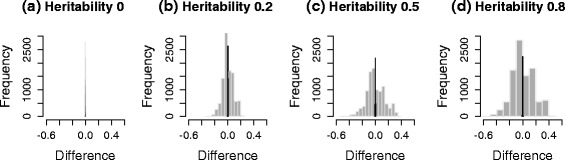
Histograms showing the differences between the covariance of statistics and the correlation of genotypes estimated from a simulated HMDP dataset. Heritability: **a** 0, **b** 0.2, **c** 0.5 and **d** 0.8. The *x*-axis represents the difference between the covariance of statistics and the correlation of genotypes, and the *y*-axis represents the frequencies. *Gray bars* represent the differences before applying genotype transformation, and *black bars* represent the differences after applying genotype transformation

**Fig. 4:**
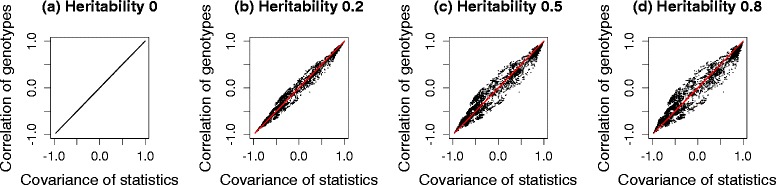
Scatter plots showing the covariance of statistics and the correlation of genotypes estimated from a simulated HMDP dataset. Heritability: **a** 0, **b** 0.2, **c** 0.5 and **d** 0.8. The *x*-axis represents the covariance of statistics, and the *y*-axis represents the corresponding correlation of genotypes. *Red* and *black dots* represent cases in which we did or did not use genotype transformation, respectively

**Fig. 5:**
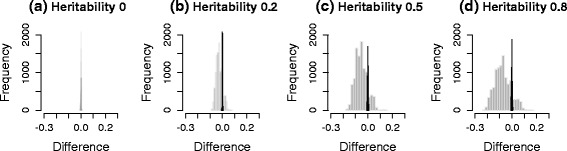
Histograms showing the differences between the covariance of statistics and the correlation of genotypes estimated from a simulated yeast dataset. Heritability: **a** 0, **b** 0.2, **c** 0.5 and **d** 0.8. The *x*-axis represents the difference between the covariance of statistics and the correlation of genotypes, and the *y*-axis represents the frequencies. *Gray bars* represent the differences before applying genotype transformation, and *black bars* represent the differences after applying genotype transformation

**Fig. 6:**
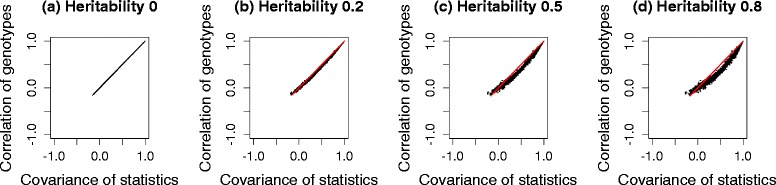
Scatter plots showing the covariance of statistics and the correlation of genotypes estimated from a simulated yeast dataset. Heritability: **a** 0, **b** 0.2, **c** 0.5 and **d** 0.8. The *x*-axis represents the covariance of statistics, and the *y*-axis represents the corresponding correlation of genotypes. *Red* and *black dots* represent cases in which we did or did not use genotype transformation, respectively

**Fig. 7:**
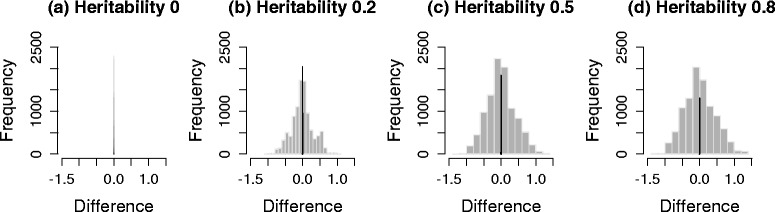
Histograms showing the differences between the covariance of statistics and the correlation of genotypes estimated from a simulated HapMap dataset. Heritability: **a** 0, **b** 0.2, **c** 0.5 and **d** 0.8. The *x*-axis represents the difference between the covariance of statistics and the correlation of genotypes, and the *y*-axis represents the frequencies. *Gray bars* represent the differences before applying genotype transformation, and *black bars* represent the differences after applying genotype transformation

**Fig. 8:**
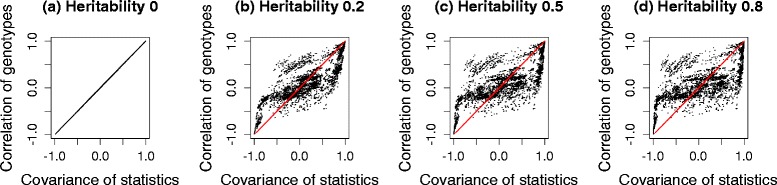
Scatter plots showing the covariance of statistics and the correlation of genotypes estimated from a simulated HapMap dataset. Heritability: **a** 0, **b** 0.2, **c** 0.5 and **d** 0.8. The *x*-axis represents the covariance of statistics, and the *y*-axis represents the corresponding correlation of genotypes. *Red* and *black dots* represent cases in which we did or did not use genotype transformation, respectively

We generated three sets of genotypes, with 100 markers each from the HMDP dataset, a yeast dataset and the HapMap dataset. Then, 10^5^ phenotypes were simulated for four different cases, each with heritability, 0, 0.2, 0.5 and 0.8. $({\hat {\beta }}/{\hat {\sigma }}) \sqrt {N}$ was used as the test statistic. We compared the correlation of the genotypes and covariance of the test statistics before and after applying the transformation strategy. The term heritability is defined as ${\sigma _{g}^{2}}/\left ({\sigma _{g}^{2}}+{\sigma _{e}^{2}}\right)$, which represents the fraction of variance explained by population structure [47], more precisely, the fraction of variance explained by all genetic variants included in calculating the kinship matrix, **K**. Figure 3 shows histograms of the differences between the covariance of test statistics and the correlation of genotypes, estimated from a simulated dataset of HMDP. Gray bars represent the differences between the covariance of test statistics and the correlation of untransformed genotypes, *r*_*ij*_. Black bars represent the differences between the covariance of test statistics and the correlation of genotypes transformed by the square root of $\hat {V}^{-1/2}$, $r^{M}_{ij}$. As shown in Fig. 3, the difference is centered at zero when we use transformed genotypes, regardless of heritability. However, if we do not use transformation, the difference deviates widely from zero as the heritability increases, indicating that the naive genotype correlation cannot effectively approximate the covariance of statistics well. Figure 4 shows scatter plots of the covariance of test statistics (*x*-axis) and the correlation of genotypes (*y*-axis). Red and black dots represent cases in which we did or did not use genotype transformation, respectively. When heritability is zero (Figs. 3a and 4a), the equality in Eq. 2 holds as expected. However, as the heritability increases (Figs. 3b–d and 4b–d), the discrepancy between the covariance of statistics and the correlation of genotypes increases. After applying our genotype transformation and using Eq. 5 to approximate the covariance of statistics, the differences are calibrated back to zero. We applied the same strategy to simulated datasets from the yeast data (Figs. 5 and 6) and HapMap data (Figs. 7 and 8), and obtained consistent results across the three species.

### MultiTrans accurately corrects for multiple testing

We examined the accuracy of our method, MultiTrans, for multiple testing in LMM. We compared MultiTrans with three different methods: Bonferroni correction; SLIDE [24], which is one of the MVN-based multiple testing correction method; and the standard parametric bootstrapping approach.

Due to the computational cost of parametric bootstrapping, we applied each method only to chromosome 1 of the HMDP dataset. Table 1 shows the per-marker thresholds of different methods at the 5 % significance level. We simulated four different situations, each with heritability 0, 0.2, 0.5 and 0.8. Across the range of heritabilities, MultiTrans yielded very accurate per-marker thresholds very close to those of parametric bootstrapping. On the other hand, the Bonferroni correction gave very stringent thresholds. Previous studies showed that SLIDE closely approximates the permutation test and gives accurate per-marker thresholds for the standard linear model [24]. When the simulated heritability is zero, LMM is equivalent to the standard linear model. Thus, it is not surprising that SLIDE gives a per-marker threshold of 6.59E-05, very close to the threshold obtained from parametric bootstrapping, 6.71E-05. However, SLIDE performed worse as the heritability increased. This is expected based on the results in the previous section showing that the discrepancy between the covariance of statistics and the correlation of genotypes increases as the heritability increases if we do not account for phenotype correlations specific to LMM.

**Table 1:** Per-marker thresholds at the 5 % significance level for different simulated heritabilities of 0, 0.2, 0.5 and 0.8, applied to chromosome 1 of the HMDP dataset

Heritability	Bonferroni	SLIDE	MultiTrans	Bootstrapping
0	5.19E-06	6.59E-05	6.59E-05	6.71E-05
0.2	5.19E-06	6.59E-05	5.17E-05	5.29E-05
0.5	5.19E-06	6.59E-05	4.71E-05	4.85E-05
0.8	5.19E-06	6.59E-05	4.54E-05	4.48E-05

### Per-marker threshold depends on both heritability and genetic relatedness

We applied MultiTrans to various datasets from different species and with different heritabilities to see how heritability affects the per-marker thresholds, as well as how the per-marker threshold changes in a dataset-specific manner. Due to the computational cost of parametric bootstrapping, in the previous section (Table 1) we tested each method only on chromosome 1, which contains 9629 markers. Taking advantage of the efficiency of MultiTrans, in this experiment we were able to apply MultiTrans to the whole genome in large datasets.

**Fig. 9:**
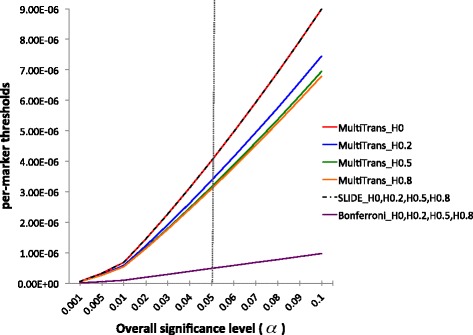
Per-marker thresholds for different heritabilities applied to the whole genome of the HMDP dataset. The *x*-axis represents the overall significance level, *α*, from 0.1. to 10 %. The *y*-axis represents the corresponding per-marker thresholds. The *gray vertical line* shows the significance level, 5 %. The *red*, *blue*, *green* and *orange solid lines* show the result of MultiTrans when heritability is 0, 0.2, 0.5 and 0.8. The *purple solid line* shows the results of Bonferroni correction for all four heritabilities. The *black dash-dot line* shows the result of SLIDE for all four heritabilities

Figure 9 shows the per-marker thresholds of the whole genome of the HMDP dataset estimated from MultiTrans for four simulated situations, each with heritability 0, 0.2, 0.5 and 0.8, over a range of significance levels from 0.1 to 10 %. The red, blue, green and orange solid lines show the per-marker thresholds of MultiTrans, and they demonstrate how heritability affected the per-marker thresholds for the HMDP dataset; as the heritability increased the per-marker thresholds decreased. However, this was not reflected in the previous methods, the Bonferroni correction (purple solid line in Fig. 9) and SLIDE (black dash-dot line in Fig. 9), whose per-marker thresholds did not change as the heritability changed.

In addition, we applied MultiTrans to the whole genome of yeast and HapMap datasets. Table 2 shows the per-marker thresholds at a significance level of 5 %, estimated from MultiTrans for the HMDP, yeast and HapMap datasets. For each dataset, four different heritabilities (0, 0.2, 0.5 and 0.8) were simulated. For all datasets, the per-marker threshold decreased as the heritability increased. However, the amount that heritability affected the per-marker thresholds differed across the datasets. As heritability changed, the HMDP and yeast datasets exhibited larger differences in their per-marker thresholds than the HapMap dataset.

**Table 2:** Per-marker thresholds at a 5 % significance level estimated from MultiTrans for different simulated heritabilities of 0, 0.2, 0.5 and 0.8, applied to the whole genome HMDP, yeast and HapMap datasets

	Dataset		
Heritability	HMDP	Yeast	HapMap
0	4.03E-06	5.09E-05	7.29E-08
0.2	3.38E-06	4.65E-05	7.08E-08
0.5	3.16E-06	4.24E-05	7.07E-08
0.8	3.10E-06	3.87E-05	7.06E-08

**Fig. 10:**
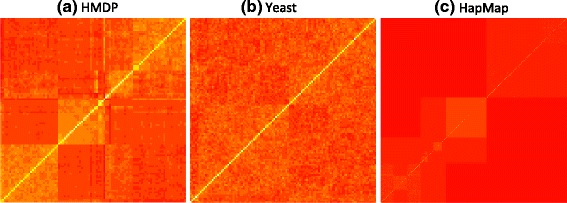
Heat maps of genetic relatedness reflected in a kinship matrix for different datasets. **a** HMDP, **b** yeast and **c** HapMap. Individuals are ordered from *left* to *right* on the *x*-axis, and from *bottom* to *top* on the *y*-axis. Each *pixel* of the heat map shows the strength of the correlation between the individuals, with *yellow* indicating strong correlation and *red* indicating no correlation

**Fig. 11:**
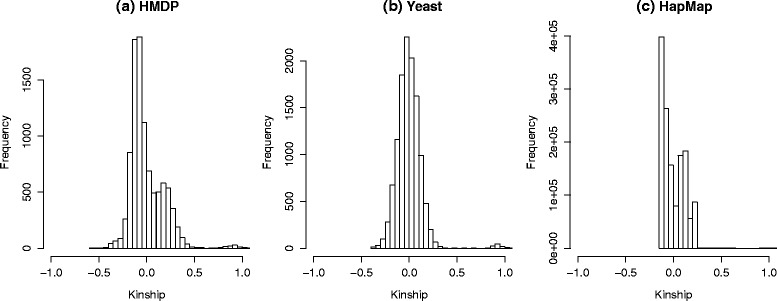
Histograms of off-diagonal values of kinship matrix. **a** HMDP, **b** yeast and **c** HapMap

The reason that different datasets show different changes in per-marker threshold given the same changes in heritability is that not only the heritability but also the amount of genetic relatedness in genotypes may affect the per-marker thresholds. For example, if individuals are less related in a study, even for a trait that is highly heritable, the correlation of genotypes, *r*_*ij*_ (Eq. 2) and the correlation of transformed genotypes, $r_{ij}^{M}$ (Eq. 5), may not show a big difference. This is because their kinship matrix **K** may be similar to the identity matrix **I**, and $\hat {V}=\hat {\sigma }_{g}^{2}\mathbf {K}+\hat {\sigma }_{e}^{2}\mathbf {I} \approx \left (\hat {\sigma }_{g}^{2}+ \hat {\sigma }_{e}^{2}\right)\mathbf {I}$, therefore, the transformation with $\hat {V}^{1/2}$ may not significantly change the correlation between the genotypes. In this case, the influence of heritability $\left (\hat {\sigma }_{g}^{2}\right)$ on the per-marker thresholds may be small. Figure 10 shows heat maps of genetic relatedness reflected in kinship matrices for the HMDP, yeast and HapMap datasets. The color of each pixel represents the strength of the relatedness, with yellow indicating strong correlation between individuals and red indicating no relatedness. Compared to the HDMP and yeast datasets, the HapMap dataset shows smaller relatedness between the individuals. In addition, we show histograms of the off-diagonal values of the kinship matrices for the HMDP, yeast and HapMap datasets (Fig. 11). The figure shows that the individuals in HapMap are related but less related to each other compared to those in the HMDP and yeast datasets. These explain that even though the per-marker thresholds are different for different heritability cases in HapMap data, their differences are less dramatic than those of HMDP data.

### MultiTrans applied to the real traits

Because MultiTrans is efficient and accurate, we were able to apply MultiTrans to a large number of real phenotypes in the HMDP, yeast and HapMap datasets. As described above, these datasets have different genetic relatedness, and the phenotypes in each dataset have different heritabilities; therefore, each phenotype will have a unique per-marker threshold. Table 3 confirms that multiple phenotypes in the three datasets have different per-marker thresholds.

**Table 3:** Per-marker thresholds for various real phenotypes of HMDP, yeast and HapMap datasets estimated from MultiTrans

HMDP		
Phenotype	Heritability	MultiTrans
Thioglycolate treated	0.036	3.80E-06
Free fluid	0.653	3.12E-06
Low-density lipoprotein	0.706	3.11E-06
Yeast		
ProbeID	Heritability	MultiTrans
YMR073C	0.010	5.06E-05
YMR242C	0.111	4.82E-05
YLR447C	0.214	4.63E-05
YDR186C	0.310	4.48E-05
YHL012W	0.409	4.34E-05
YOL144W	0.503	4.23E-05
YFL018C	0.615	4.09E-05
YCR107W	0.700	3.99E-05
YMR312W	0.819	3.85E-05
YNL046W	0.911	3.73E-05
HapMap		
ProbeID	Heritability	MultiTrans
ILMN 1756694	0.013	7.11E-08
ILMN 1851657	0.156	7.06E-08
ILMN 1803219	0.225	7.05E-08
ILMN 1741165	0.401	7.04E-08
ILMN 1704746	0.728	7.02E-08

### Efficiency of MultiTrans

**Fig. 12:**
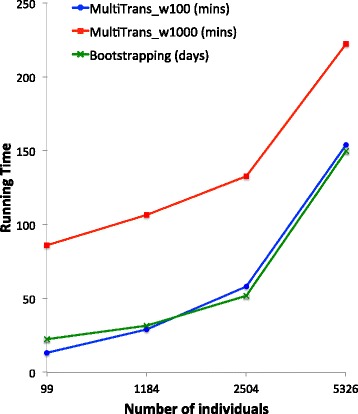
Comparison of running time of MultiTrans and the parametric bootstrapping. The running times evaluated for 100,000 markers and 10,000 samplings. The *x*-axis shows the number of individuals, and the *y*-axis shows the running time. The *blue* and *red lines* show the running times of MultiTrans using window sizes of 100 and 1000, respectively, in minutes. The *green line* shows the running time of parametric bootstrapping in days

To demonstrate the efficiency of MultiTrans, we compared the running time of MultiTrans and parametric bootstrapping, which can accurately correct *p* values for multiple testing in LMM. Both MultiTrans and the parametric bootstrapping must calculate the inverse square root of the covariance matrix $\hat {V}^{-1/2}$ once. However, parametric bootstrapping needs to sample null phenotypes from MVN multiple times and estimate statistics for each of them, which takes a lot of time [31, 35]. To compare the running time of MultiTrans and parametric bootstrapping, we estimated the running time of both methods utilizing four different datasets; HMDP [11], HapMap [42], 1000Genomes [48] and NFBC (Northern Finland Birth Cohorts) [49], which contains 99, 1184, 2504 and 5326 individuals, respectively. MultiTrans assumes local linkage disequilibrium and that the statistics outside the range of a window are independent of each other. It applies a sliding-window approach (see section “Methods” for the details of the sliding-window approach). The running time of MultiTrans depends on the size of the window, so we applied two different window sizes, 100 and 1000. Figure 12 shows the running times of MultiTrans and parametric bootstrapping for different numbers of individuals for 100,000 markers. For both MultiTrans and the parametric bootstrapping, 10,000 samplings were performed, and the running times were extrapolated from one chromosome. When the number of individuals was 5326, the parametric bootstrapping took about 5 months, which is impractical, whereas MultiTrans took only 2.57 h or 3.71 h using a window size of 100 or 1000,respectively. Even for 99 individuals, parametric bootstrapping took more than 22 days, whereas MultiTrans took only 13.35 min or 1.45 h using a window size of 100 or 1000, respectively. The result shows that even for a small study, MultiTrans is 2421 times faster or 376 times faster than the parametric bootstrapping using a window size of 100 or 1000, respectively. The discrepancy between the running times of MultiTrans and parametric bootstrapping will increase not only as the number of individuals increases, but also as the numbers of samplings or markers increases (data not shown). More details of the running time are discussed in section “Methods”.

### Normality assumption in MultiTrans

Our framework is based on LMM, which assumes the normality of phenotypes. Moreover, when we derived the covariance structure of statistics (see section “Methods”), we used the *z* score statistic, which will follow a normal distribution under the normality of phenotypes. However, if phenotypes do not follow a normal distribution and are highly skewed or aggregated, first, LMM fitting might not work well, and second, the statistic might not follow a normal distribution.

Nevertheless, for many tests assuming normality of phenotypes, it is known that even if the phenotypes do not follow a normal distribution, the *p* value is approximately calibrated and therefore the corresponding *z* score follows a normal distribution. To show how this normality assumption affects the results, we computed the standard *z* scores (which assumes normality of phenotypes) from microbiome data, which does not follow a normal distribution. Additional file 1: Figure S1a shows abundances of a genus-level taxon of microbiome data [50], which apparently is not normal, and Additional file 1: Figure S1b shows the *Q*–*Q* plot of their test statistics estimated from the phenotypes. It shows that even if the phenotypes do not follow a normal distribution, the statistics approximately follows a normal distribution.

## Discussion

Multiple testing correction is a very well-studied problem in the context of GWAS [20–24, 51, 52], with the most widely utilized approach being the permutation test. In most modern GWAS, LMM is applied to account for the effect of population structure or increase statistical power. Unfortunately, in these studies, the permutation test is not only impractical due to the computational cost [23], but also the assumptions required for permutation testing are not satisfied under LMM and may lead to spurious associations.

Here, we show that the heritability of a trait affects the significance threshold, as well how to perform multiple testing correction in the context of LMM association studies. Our proposed method, MultiTrans, accurately corrects for multiple hypothesis testing and is also efficient, making it applicable to large GWAS. In addition, we demonstrated the accuracy and efficiency of MultiTrans utilizing mouse, yeast and human datasets.

In this paper, we proposed a parametric bootstrapping resampling approach to set up the gold standard approach for multiple testing in LMM. Parametric bootstrapping is consistent with the assumed model of LMMs. Some previous methods try to generate the null samples under LMM by improving the permutation test. Abney [53] proposed a method, referred to as MVNpermute, which estimates maximum likelihood estimates for LMM parameters under the assumption that phenotypes follow MVN then it permutes the residuals to generate the null samples. This method does not assume normality of phenotypes when they sample the null phenotypes by permuting the residuals. However, they estimate the residuals based on the assumption that phenotypes follow MVN to estimate the LMM parameters. Another approach was proposed by He et al. [54]. This transforms the phenotypes with the covariance matrix of phenotypes, permutes them and then transforms them back. The advantage of this approach is that it does not assume the normality of the phenotypes; thus, it is applicable for data that do not follow a normal distribution. However, the permutation test is computationally very expensive, and thus, often no more than 10^4^ permutations are used in GWAS [24, 53, 54], which is not sufficient for the significance test for GWAS datasets (Table 3). Thus, permutation-based methods are impractical for GWAS datasets.

Instead of sampling phenotypes, MultiTrans samples statistics directly from a MVN whose covariance matrix is estimated from transformed genotypes and it applies a sliding-window Monte Carlo approach to speed up the sampling procedure. Comparing the running time of MultiTrans and parametric bootstrapping, which can accurately correct the *p* values for multiple testing in LMM, we showed that the parametric bootstrapping approach is impractical even for a small study, whereas MultiTrans can dramatically reduce the running time.

Our results show that the heritability changes the covariance of statistics and per-marker thresholds. In addition, we made the novel observation that the per-marker threshold tends to decrease as the heritability increases for the HMDP, yeast and HapMap datasets. We also provided an intuition regarding how genetic relatedness in datasets affects the per-marker threshold. To our knowledge, our study is the first study to explain the relationship between heritability, genetic relatedness and the per-marker threshold.

The ideas behind our approach extend multivariate normal approaches for modeling the joint distribution of GWAS statistics to scenarios in which mixed models are utilized to compute the association statistics. In this paper, we demonstrated how this extension can be used to compute the significance threshold for multiple testing correction; however, this framework can be utilized for other applications of MVNs as well. For example, similar extensions can be applied to fine mapping methods [44, 55, 56], GWAS statistic imputation [57, 58], joint testing [59], follow-up single-nucleotide polymorphism (SNP) selection [43], etc. In frameworks utilizing MVN, one assumes that the test statistic follows a normal distribution. Since some statistical tests assume normality of phenotypes, there can be issues relating to this assumption. However, the normality of test statistics is not much affected by the normality of phenotypes, which is discussed in section “Results”. In addition, several techniques can transform the data into a normal distribution such as inverse normal transformation or WarpedLMM [60], which are heavily used by many studies [32, 50, 61–64]. Moreover, Sul et al. [65] recently applied the MVN framework to multiple testing correction in eQTL studies where the Spearman correlation statistic, a non-parametric test, was used. This study shows that MVN can be applied beyond parametric settings and can work well independently from the normality assumption. Lastly, there are a number of ways in which MultiTrans could be improved. One of which is to improve the way it calculates the kinship matrix, which is an active research area these days. More precisely, the actual heritability we are using is the variance explained by genetic variants included in the kinship matrix; thus, it is important to estimate the kinship matrix accurately. For example, we can use only SNPs that are linearly independent of the SNP that we are testing [29].

## Methods

### Previous multiple testing correction methods for non-LMM

#### Permutation test

The permutation test gives a simple way to compute the null sampling distribution for a test statistic by repeatedly permuting either genotypes or phenotypes and computing the association statistic for each permutation. The permutation test can be thought of as a resampling approach that samples individuals from a uniform distribution without replacement. The permutation test accurately accounts for the correlation structure of the genome, and therefore, has been used as the gold standard for GWAS. However, it is computationally expensive, and its running time is linearly dependent on the number of individuals.

#### Methods using multivariate normal approximation

Several previous studies proposed alternative approaches to permutation because the permutation test is computationally expensive especially when the number of individuals is large. The idea underlying these approaches is sampling of test statistics directly from MVN, taking advantage of the fact that the statistics over multiple markers asymptotically follows a MVN [21, 22].

Below, we show how to obtain the covariance matrix of the MVN. Let *m* be the number of markers, *S*_*i*_ be a statistic for the *i*th marker and *Σ*={Cov(*S*_*i*_,*S*_*j*_)} be the *m*×*m* covariance matrix between the statistics. Assuming the following linear model, we can derive the covariance matrix for the MVN:

$$Y=\mu \mathbf{1_{n}}+X_{i}\beta_{i}+\mathbf{e}.  $$

Here, *n* is the number of individuals, *μ* is a mean of the phenotypic values, 1_*n*_ is a vector of *n* ones, *Y* is a vector of length *n* with the phenotypic values, *X*_*i*_ is a vector of length *n* with the genotypic values of the *i*th marker, *β*_*i*_ is their coefficients and **e** is a vector of length *n* sampled from $\mathcal {N}\left (0, {\sigma _{e}^{2}}\mathbf {I}\right)$ accounting for the residual errors. Here, we assume that *Y* and *X*_*i*_ are normalized as mean 0 and variance 1. Then, the phenotype follows a MVN with a mean and variance as follows:

$$Y \sim \mathcal{N}\left(\mu \mathbf{1_{n}}+X_{i}\beta_{i}, {\sigma_{e}^{2}}\mathbf{I}\right).  $$

The ordinary least-squares solutions of *β* for the *i*th and *j*th markers are as follows:

$$\begin{aligned} \hat{\beta}_{i} & = \left({X_{i}^{T}}X_{i}\right)^{-1}{X_{i}^{T}}Y\sim \mathcal{N} \left(\beta_{i},\frac{{\sigma_{e}^{2}}}{{X_{i}^{T}}X_{i}}\right)\\ \hat{\beta}_{j} & = \left({X_{j}^{T}}X_{j}\right)^{-1}{X_{j}^{T}}Y\sim \mathcal{N} \left(\beta_{j},\frac{{\sigma_{e}^{2}}}{{X_{j}^{T}}X_{j}}\right). \\  \end{aligned} $$

The statistics of the two markers are computed as follows:

$$\begin{aligned} S_{i} & = \frac{\hat{\beta}_{i}}{\hat{\sigma}_{e}}\sqrt{{X_{i}^{T}}X_{i}}\sim \mathcal{N}\left(\beta_{i} \frac{\sqrt{{X_{i}^{T}}X_{i}}}{\sigma_{e}}, 1\right)\\ S_{j} & = \frac{\hat{\beta}_{j}}{\hat{\sigma}_{e}}\sqrt{{X_{j}^{T}}X_{j}}\sim \mathcal{N}\left(\beta_{j} \frac{\sqrt{{X_{j}^{T}}X_{j}}}{\sigma_{e}}, 1\right). \\  \end{aligned} $$

Here, the estimated values for *μ*, **e** and *σ* for the *i*th marker are as follows:

$$\begin{array}{*{20}l} \hat{\mu} & = \frac{\mathbf{1_{n}}^{T}X_{i}}{{X_{i}^{T}}X_{i}}, \\ \hat{\mathbf{e}} &= Y-\hat{\mu}\mathbf{1_{n}}-X\hat{\beta}\\ \end{array} $$

and

$$\begin{array}{*{20}l} \hat{\sigma} = \sqrt{\frac{\hat{\mathbf{e}}^{T}\hat{\mathbf{e}}}{n-2}}. \end{array} $$

Then, we can prove that the covariance of the two statistics, Cov(*S*_*i*_,*S*_*j*_), is equal to the correlation between the genotypes, *r*_*ij*_, as follows [24, 44, 56]:

(6) $$ \begin{aligned} \text{Cov}(S_{i}, S_{j}) &= \text{Cov}\left(\frac{\hat{\beta}_{i}}{\sigma_{e}}\sqrt{{X_{i}^{T}}X_{i}}, \frac{\hat{\beta}_{j}}{\sigma_{e}}\sqrt{{X_{j}^{T}}X_{j}}\right)\\ & = \frac{1}{{\sigma_{e}^{2}}}\text{Cov}\left(\frac{{X_{i}^{T}}Y}{\sqrt{{X_{i}^{T}}X_{i}}}, \frac{{X_{j}^{T}}Y}{\sqrt{{X_{j}^{T}}X_{j}}}\right)\\ & = \frac{{X_{i}^{T}}X_{j}}{\sqrt{{X_{i}^{T}}X_{i}}\sqrt{{X_{j}^{T}}X_{j}}}\\ & = \text{Cor}\left(X_{i}, X_{j}\right)\equiv r_{ij}.  \end{aligned}  $$

Previous studies showed that this relationship between genotype correlation and MVN covariance holds for binary traits as well, using different methods of derivation [22, 24].

Using the properties of Eq. 6, we can sample the statistics directly from the MVN with mean 0 and variance *Σ*={*r*_*ij*_} instead of permuting phenotypes. In fact, in this sampling, phenotype information is not needed. Specifically, under the null hypothesis, by the multivariate central limit theorem [45], if the number of individuals, *n*, is large, the vector of statistics (*S*_1_,…,*S*_*m*_) asymptotically follows a MVN with mean 0 and variance *Σ*. Given a pointwise *p* value *u*, let *R*(*u*) be the *m*-dimensional rectangle with corners *Φ*^−1^(*u*/2)**1**_*m*_ and *Φ*^−1^(1−*u*/2)**1**_*m*_, where *Φ* is the cumulative density function of the standard normal distribution and **1**_*m*_ is the vector of *m* ones. Then, the significance level *p*_*α*_ is approximated as the outside-rectangle probability as shown in Fig. 1,

(7) $$ p_{\alpha} = 1- {\frac{1}{(2\pi)^{\frac{m}{2}}|\Sigma|^{\frac{1}{2}}}}\int_{R_{(u)}}\mathrm{e}^{-\frac{1}{2}X^{T}\Sigma^{-1}X_{dX}}.   $$

Thus, given an overall significance threshold *α*, the per-marker threshold can be approximated by searching for a pointwise *p* value *u* whose significance level *p*_*α*_ is *α*.

### Multiple testing correction methods for LMM

#### Parametric bootstrapping resampling approach

We first set up the gold standard approach of multiple testing in LMM, which is the equivalent of the permutation test for LMM. We emphasize that the traditional permutation test and its variations do not work for LMM. The idea underlying permutation testing is that each permutation is a sample from the null distribution, which is not the case in LMM, because the permutation alters the dependency of the phenotype on the relatedness structure. If we permute phenotypes, the relatedness structure between the individuals and its effect on phenotype are ignored, which can lead to an inflation of *p* values.

**Fig. 13:**
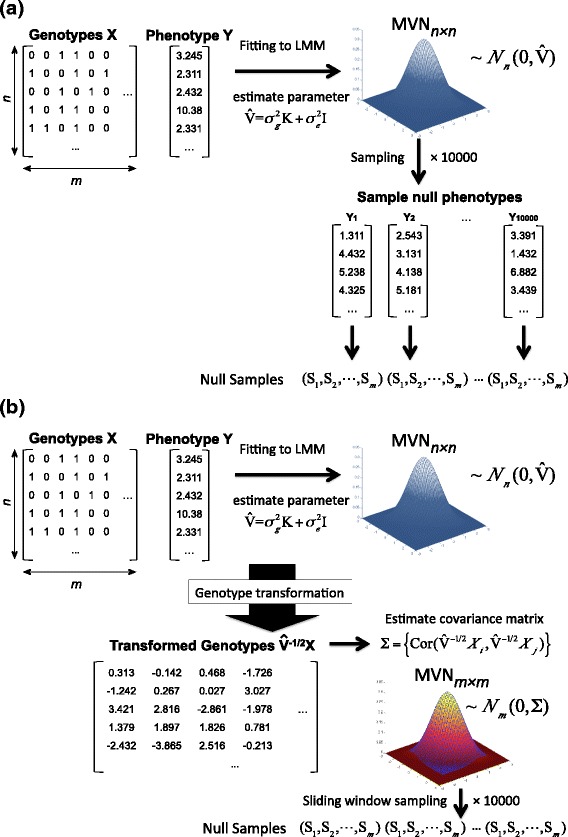
Overview of the resampling procedures. **a** Parametric bootstrapping and **b** MultiTrans, with 10^4^ sampling applied for both parametric bootstrapping and MultiTrans

We propose a resampling-based multiple hypothesis testing approach for LMM, which utilizes the parametric bootstrapping strategy. Figure 13a shows an overview of the parametric bootstrapping applied to multiple hypothesis testing. It is described as follows. First, by fitting to LMM, we estimate parameters $\hat {\sigma _{g}}^{2}$ and $\hat {\sigma }_{e}^{2}$ to generate a covariance matrix of the data, $\hat {V}=\hat {\sigma }_{g}^{2}\mathbf {K}+\hat {\sigma }_{e}^{2}\mathbf {I}$. Second, we sample size-*n* vectors of null phenotypes from the distribution from MVN with the covariance matrix $\hat {V}$. Third, using each size-*n* vector of those null phenotypes, we compute null statistics (*S*_1_,*S*_2_,…,*S*_*m*_). This parametric bootstrapping approach can be thought of as the permutation-equivalent for LMM. Similar approaches were used in previous studies [46, 53, 54], some of which are discussed in Additional file 1. Unfortunately, this parametric bootstrapping approach is computationally very expensive.

#### MultiTrans

##### MVN approximation for LMM

As described in the previous section, the parametric bootstrapping strategy is impractical due to its high computational cost. To make the procedure efficient, we propose a new approach, MultiTrans. MultiTrans alternatively samples statistics directly from MVN without needing to generate any null phenotypes. Figure 13b shows an overview of MultiTrans. Once we obtain the null samples, we can obtain the per-marker threshold using Eq. 7. However, the challenge is to characterize the covariance of MVN for LMM.

##### Covariance of MVN in LMM

For LMM, Eq. 6 is no longer valid. That is, we cannot use the genotype correlation matrix as the covariance matrix of MVN for LMM.

To derive the covariance matrix, we assume a LMM instead of the linear model as follows:

$$Y=\mu\mathbf{1_{n}}+X_{i}{\beta_{i}^{M}}+\mathbf{g}+\mathbf{e},  $$

where *μ* is the mean of the phenotypic values, 1_*n*_ is a vector of *n* ones, *Y* is a vector of length *n* with the phenotypic values, *X*_*i*_ is a vector of length *n* with the genotypic values of the *i*th marker, ${\beta _{i}^{M}}$ is their coefficients under the LMM, **g** is a vector of length *n* sampled from $\mathcal {N}\left (0, {\sigma _{g}^{2}}\mathbf {K}\right)$ accounting for population structure effects where **K** is a *n*×*n* matrix that explains the correlation between the individuals induced by population structure, and **e** is a vector of length *n* sampled from $\mathcal {N}(0, \sigma ^{2}\mathbf {I})$ accounting for the residual errors. Under this model, the phenotype follows a MVN with a mean and variance as follows:

$$Y \sim \mathcal{N}\left(\mu\mathbf{1_{n}}+X_{i}{\beta_{i}^{M}}, {\sigma_{g}^{2}}\mathbf{K}+{\sigma_{e}^{2}}\mathbf{I}\right).  $$

Given the observed data, it is straightforward to fit LMM and estimate parameters ${\sigma ^{2}_{g}}$ and ${\sigma ^{2}_{e}}$ using standard strategies, which define the covariance matrix of phenotypes, $\text {Cov}(Y) = \hat {V}= \hat {\sigma }_{g}^{2}\mathbf {K}, +\hat {\sigma }_{e}^{2}\mathbf {I}$. Now we utilize the fact that after obtaining $\hat {V}$, the remaining regression procedure is equivalent to performing ordinary least-squares in the transformed space,

$$\hat{V}^{-1/2}Y \sim \mathcal{N}\left(\hat{V}^{-1/2}\mu\mathbf{1_{n}}+\hat{V}^{-1/2}X_{i}{\beta_{i}^{M}}, \mathbf{I}\right),  $$

where both genotypes and phenotypes are transformed by a factor $\hat {V}^{-1/2}$. Assuming that $\hat {V}^{-1/2}X_{i}$ and $\hat {V}^{-1/2}Y$ are normalized as mean 0 and variance 1 (without loss of generality), the ordinary least-squares solutions of ${\beta _{i}^{M}}$ for the *i*th marker and *j*th marker are as follows:

$${} {{\begin{aligned} \hat{\beta}_{i}^{M} &= \left({X_{i}^{T}}\hat{V}^{-1}X_{i}\right)^{-1}{X_{i}^{T}}\hat{V}^{-1}Y\sim\mathcal{N}\left({\beta^{M}_{i}},\left({X_{i}^{T}}\hat{V}^{-1}X_{i}\right)^{-1}\right)\\ \hat{\beta}_{j}^{M} &= \left({X_{j}^{T}}\hat{V}^{-1}X_{j}\right)^{-1}{X_{j}^{T}}\hat{V}^{-1}Y\sim\mathcal{N}\left({\beta^{M}_{j}},\left({X_{j}^{T}}\hat{V}^{-1}X_{j}\right)^{-1}\right).\\ \end{aligned}}} $$

The statistics are computed as follows:

$$\begin{aligned} S_{i} & = \hat{\beta}_{i}^{M}\sqrt{{X_{i}^{T}}\hat{V}^{-1}X_{i}}\sim \mathcal{N}\left({\beta_{i}^{M}}\sqrt{{X_{i}^{T}}\hat{V}^{-1}X_{i}}, 1\right)\\ S_{j} & = \hat{\beta}_{j}^{M}\sqrt{{X_{j}^{T}}\hat{V}^{-1}X_{j}}\sim \mathcal{N}\left({\beta_{i}^{M}}\sqrt{{X_{j}^{T}}\hat{V}^{-1}X_{j}}, 1\right).\\ \end{aligned} $$

Accordingly, the correlation between the statistics changes from Eq. 6 to the following where the correlation between the statistics are equal to the correlation between the marker transformed by the inverse square root of $\hat {V}$:

$${} {{\begin{aligned} \text{Cov}\left({S_{i}^{M}}, {S_{j}^{M}}\right) &= \text{Cov}\left(\frac{{X_{i}^{T}}\hat{V}^{-1}Y}{\sqrt{{X_{i}^{T}}\hat{V}^{-1}X_{i}}}, \frac{{X_{j}^{T}}\hat{V}^{-1}Y}{\sqrt{{X_{j}^{T}}\hat{V}^{-1}X_{j}}}\right)\\ & = \frac{{X^{T}_{i}}\hat{V}^{-1/2}\left(\hat{V}^{-1/2}\right)^{T}X_{j}}{\sqrt{{X^{T}_{i}}\left(\hat{V}^{-1/2}\right)^{T}\hat{V}^{-1/2}X_{i}}\sqrt{{X^{T}_{j}} \left(\hat{V}^{-1/2}\right)^{T}\hat{V}^{-1/2}X_{j}}}\\ & = \text{Cor}\left(\hat{V}^{-1/2}X_{i}, \hat{V}^{-1/2}X_{j}\right)=r^{M}_{ij}.  \end{aligned}}} $$

Utilizing the covariance matrix estimated from transformed genotypes, we can generate a large number of samples, (*S*_1_,*S*_2_,…,*S*_*m*_), to approximate MVN and correct *p* values by integrating over the outside of the rectangle, as in Eq. 7.

##### Sliding-window approach

If *m* is large, the standard sampling approach that samples (*S*_1_,*S*_2_,…,*S*_*m*_) from MVN using Cholesky decomposition [66] is computationally very expensive. In our approach, we assume that there is no correlation between statistics at loci that are far apart in the genome after correcting for population structure. We term this assumption the local linkage disequilibrium assumption. We first note that this is a conservative assumption and cannot lead to false positives. By ignoring possible linkage disequilibrium results in possibly more conservative significance thresholds. Since the driver of linkage disequilibrium between distant loci and the correlation between statistics at these loci is population structure itself, it is natural to assume that after correction, the statistics will no longer be correlated. Thus, it is both appropriate and conservative to make the local linkage disequilibrium assumption. Under the local linkage disequilibrium assumption, the statistics at distant markers are uncorrelated and one can split the region into small blocks to decrease computational cost dramatically. Many previous methods [22, 23] used a block-wise strategy; however, they are known to lead to overly conservative estimates by ignoring the inter-block correlations [24]. Thus, we perform a sliding-window approach as follows to incorporate the inter-block correlations to estimate the *p* values accurately [24]. Let *f*(*S*_1_,*S*_2_,…,*S*_*m*_) be the joint probability density function of the statistics. Under the local linkage disequilibrium assumption, the statistics at distant markers are uncorrelated. Thus, given a window size *w*, we can assume that *S*_*i*_ is conditionally independent of *S*_1_,*S*_2_,…,*S*_*i*−*w*−1_ given *S*_*i*−*w*_,*S*_*i*−*w*+1_,…,*S*_*i*−1_. Utilizing the chain rule,

$$\begin{aligned} &f(S_{1}, S_{2}, \dots,S_{m}) = f(S_{1})f(S_{2}|S_{1})f(S_{3}|S_{1},S_{2}) \dots\\& f(S_{m}|S_{m-w}, \dots, S_{m-1}). \end{aligned}  $$

Thus, we can sample *S*_*i*_ given *S*_*i*−*w*_,*S*_*i*−*w*+1_,…,*S*_*i*−1_, based on the conditional distribution *f*(*S*_*i*_|*S*_*i*−*w*_,…,*S*_*i*−1_) and efficiently generate a large number of samples.

#### Running time of parametric bootstrapping and MultiTrans

Both parametric bootstrapping and MultiTrans require fitting the data to LMM to estimate the variance components of LMM, *σ*_*g*_ and *σ*_*e*_, estimating the inverse square root of the covariance matrix, $\hat {V}^{-1/2}$, and transforming the genotypes, which takes *O*(*n*^3^+*n*^2^*m*) where *n* is the number of individuals and *m* is the number of markers. The most computationally expensive step of both of the methods is the sampling process, which causes the main difference in running time between the two. For parametric bootstrapping, we need to sample null phenotypes from MVN with *n*×*n* covariance matrix $\hat {V}$, which takes *O*(*n*^3^). Then we calculate the test statistic using LMM. We can reduce the time for calculating the test statistic by using pre-estimated $\hat {V}^{-1/2}$ to transform the sampled phenotypes (*O*(*n*^2^)) and using pre-computed transformed genotypes, $\hat {V}^{-1/2}X$. However, we still need to perform the simple linear regression on the transformed genotypes and sampled phenotypes, which takes *O*(*nm*). Thus, the total complexity excluding LMM fitting is *O*(*s*(*n*^3^+*n*^2^+*nm*)) where *s* is the number of repeats. On the other hand, MultiTrans needs only to estimate the covariance matrix of the transformed genotypes, which takes *O*(*nm*^2^), and to sample statistics directly from MVN with *m*×*m* covariance matrix, which can be performed efficiently using the sliding-window approach described in section “Sliding-window approach”. This could be done in *O*(*w*^3^*m*), where *w* is the window size used in the sliding-window approach. As a result, we can reduce the sampling process of parametric bootstrapping, *O*(*s*(*n*^3^+*n*^2^+*nm*)), into *O*(*sw*^3^*m*). We note that the time complexity of each step could be reduced using various special mathematical techniques [27, 29, 31, 67–69].

### HMDP dataset

We evaluated our approach using a HMDP (high-resolution association mapping) mouse dataset [11] that contains 102,987 SNPs from 99 individuals. SNPs with a minor allele frequency less than 5 % and missing more than 10 % are filtered. To test the difference between the covariance of test statistics and the correlation between the genotypes, we generated a simulated dataset by extracting 100 SNPs from chromosome 1. Seven phenotypes with different heritabilities, which were estimated from the HMDP dataset [11], were used for section “MultiTrans applied to the real traits”.

### Microbiome dataset

To show how our normality assumption of phenotypes affects the results of test statistics, we computed test statistics using a gut microbiome dataset from 592 mice from 110 HMDP strains, which does not follow a normal distribution [50]. The study protocol has been described in detail elsewhere [70]. Bacterial 16S rRNA gene V4 region was sequenced using an Illumina MiSeq platform and the data were analyzed using established guidelines [71]. The relative abundance of each taxon was calculated by dividing the sequences pertaining to a specific taxon by the total number of bacterial sequences for that sample. We focused on abundant microbes, operational taxonomic units with at least 0.01 % relative abundance and for the GWAS, we used 197,885 SNPs and a genus-level taxon. Minor allele frequency less than 5 % and missing values more than 10 % were filtered out.

### Yeast dataset

We evaluated our approach utilizing a yeast dataset [10] that contains 2956 SNPs in 109 segregants. To test the difference between the covariance of test statistics and the correlation between the genotypes, we generated a simulated dataset by extracting 100 consecutive SNPs from chromosome 4. Ten gene expressions with different heritabilities, which were estimated from the yeast dataset [10], were used for section “MultiTrans applied to the real traits”.

### HapMap dataset

We evaluated our approach utilizing a HapMap Phase 3 dataset [42] that contains 1,070,114 SNPs from 1184 individuals. SNPs with a minor allele frequency less than 5 % and missing more than 10 % are filtered. To test the difference between the covariance of test statistics and the correlation between the genotypes, we generated a simulated dataset by extracting 100 consecutive SNPs from chromosome 22. Five gene expressions with different heritabilities, which were estimated from the HapMap dataset [42], were used for section “MultiTrans applied to the real traits”.

### Data availability

The HMDP dataset [11] is available from the Gene expression omnibus (GEO) under accession number GSE16780, the microbiome dataset [50] is available from the Sequence Read Archive (SRA) under accession number SRP059760, the yeast dataset [10] is available from GEO under accession number GSE9376, and the HapMap Phase 3 dataset [42] is available at http://hapmap.ncbi.nlm.nih.gov/.

### Implementation

For the MultiTrans results in section “MultiTrans accurately approximates covariance between test statistics” (Table 1) and section “Per-marker threshold depends on both heritability and genetic relatedness” (Fig. 9), a window size of 1000 was used and 10^7^ samplings were performed. For the parametric bootstrapping results in section “MultiTrans accurately approximates covariance between test statistics” (Table 1), 10^5^ samplings were performed. To evaluate our method for various ranges of heritabilities, we applied it for four different heritabilities, 0, 0.2, 0.5 and 0.8. The kinship matrix was estimated using all the SNPs in each dataset. However, several techniques can estimate a kinship matrix [29] and our approach can be used for kinship matrices computed in any way and it will give the multiple testing significance threshold for a model assuming the corresponding kinship matrix. To estimate *p* values and the variance components (${\sigma _{g}^{2}}$ and ${\sigma _{e}^{2}}$) for LMM, an LMM solver, pylmm [72] was used. In practice, however, other LMM-based methods, such as EMMA [26], EMMAX [27], FaST-LMM [29], etc., could be also used.

### Ethics approval

No ethics approval was required for the study.

### Software availability and license

The software and the source code are available at https://sourceforge.net/projects/multitrans/files/. The installation package and instructions are available at http://genetics.cs.ucla.edu/multiTrans/. MultiTrans is offered under the GNU Affero GPL, Version 3 (AGPL-3.0). For details of the license, see https://www.gnu.org/licenses/why-affero-gpl.html.

## Conclusions

Multiple hypothesis testing is an essential step in GWAS analysis. Although the correct per-marker threshold differs as a function of species, marker densities, genetic relatedness, and trait heritability, no previous multiple testing correction methods can comprehensively account for these factors. In this paper, we describe MultiTrans, an efficient and accurate multiple testing correction approach for linear mixed models. Our method performs a unique transformation of genotype data to account for genetic relatedness and heritability under linear mixed models, as well as to efficiently utilize the multivariate normal distribution. We were able to estimate per-marker thresholds as accurately as the gold standard approach applying to mouse, yeast, and human datasets, while reducing the time required from months to hours. We further provide an intuition about the relationships between per-marker threshold, genetic relatedness, and heritability, based on our observations in real data.

## Additional file

Additional file 1 Supplementary Figure, **Figure S1.** Distribution of phenotypes and corresponding statistics from microbiome data. (PDF 951 kb)
