# Supplementary material for: Multiple testing correction in linear mixed models
Source: Genome Biol. 2016 Apr 1;17:62. doi: 10.1186/s13059-016-0903-6 (PMC4818520; doi:10.1186/s13059-016-0903-6)
Supplement: Additional file 1 — Supplementary Figure, Figure S1. Distribution of phenotypes and corresponding statistics from microbiome data. (PDF 951 kb) [file 13059_2016_903_MOESM1_ESM.pdf]

Supplementary Figure

Multiple testing correction in linear mixed models

Joo et al.

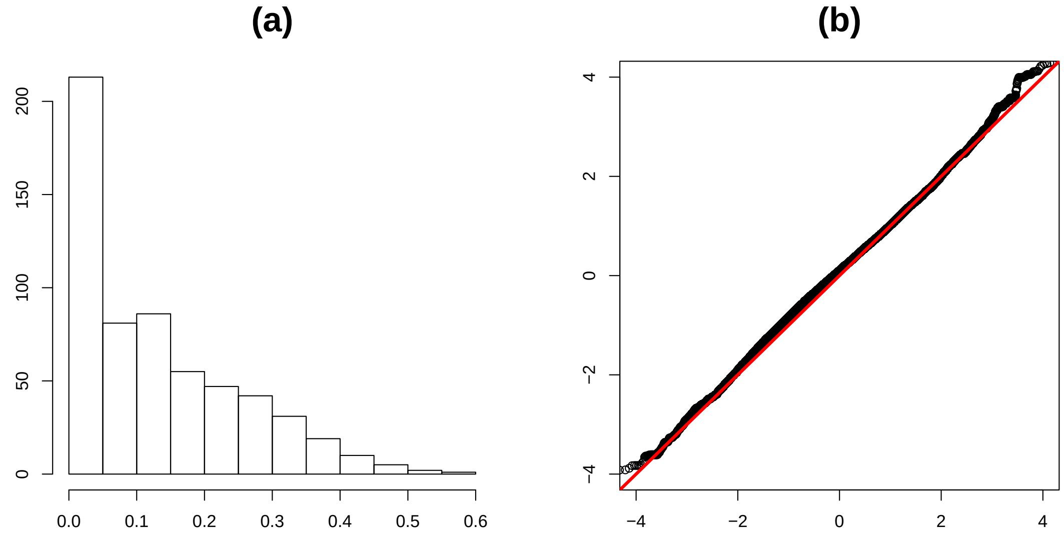

Figure S1. Distribution of phenotypes and corresponding statistics from microbiome data. (a) Histogram of phenotype values. The x-axis shows the phenotype values and the y-axis shows the frequencies. (b) Quantile plot of statistics estimated from the phenotypes shown in (a). The x-axis shows the quantile of a normal distribution and the y-axis shows the quantile of the corresponding z-scores. The red line shows a diagonal line.
